# Supplementary material for: Sigma-1 Receptor Promotes Glycolysis in Neuronal Systems by Suppressing GRIM19
Source: bioRxiv. 2025 Jul 31:2025.07.28.667250. Preprint. [Version 1] doi: 10.1101/2025.07.28.667250 (PMC12324332; doi:10.1101/2025.07.28.667250)

bioRxiv preprint doi: https://doi.org/10.1101/2025.07.28.667250; this version posted July 31, 2025. The copyright holder for this preprint (which was not certified by peer review) is the author/funder. This article is a US Government work. It is not subject to copyright under 17 USC 105 and is also made available for use under a CC0 license.

Supplementary Fig1. ***S1R overexpression rescues energy metabolism change.*** A- Transiently transfected N2a cells (up : KO GFP, down: KO S1RGFP). B-%PER glycolysis. C-MitoOCR/glycoPER. D- Percentage of positive cells. E-Stabilized S1R-overexpressing N2a cells (up : KO GFP, down : KO S1RGFP);. F-%PER glycolysis. G-MitoOCR/glycoPER. H- Percentage of positive cells. I- (top) Enolase signal intensity compared to Wt; (bottom) Western blot and total protein staining. J- (top) GRIM19 signal intensity compared to Wt; (bottom) Western blot and total protein staining. K- (top) PDH signal intensity compared to Wt; (bottom) Western blot and total protein staining. L- (top) LDH signal intensity compared to Wt; (bottom) Western blot and total protein staining. Statistical analysis B, C, F, G, I, J, K and L One-way ANOVA followed by multiple comparisons (* $p < 0.05$, ** $p < 0.01$, *** $p < 0.001$)

bioRxiv preprint doi: https://doi.org/10.1101/2025.07.28.667250; this version posted July 31, 2025. The copyright holder for this preprint (which was not certified by peer review) is the author/funder. This article is a US Government work. It is not subject to copyright under 17 USC 105 and is also made available for use under a CC0 license.

Supplementary Fig2. ***NAD/NADH-Glo™ and Peredox.*** A-Schema highlighting the NAD^+/NADH uptake assay and its use with N2a cells and primary cortical neurons. B- Signal intensity of SLC25A13 compared to that of Wt protein level. C- Signal intensity of NMNAT3 compared to that of Wt protein level. D- Video of Peredox fluorescent intensity changes with an exogenous application of lactate and pyruvate. Statistical analysis was one-way ANOVA followed by multiple comparisons (*$p < 0.05$)

Supplementary Fig3. ***Glucose Uptake-Glo™.*** A-Schema highlighting the Glucose Uptake assay and its use with N2a cells.

Supplementary Fig.4 ***RT-qPCR and mRNA in ribosomal fractions.*** A- Relative *Grim19* mRNA expression in A- N2a cells and in B- primary culture of cortical neurons. C- *Grim19* mRNA distribution across ribosomal fractions. Statistical analysis for A and B: Student's t-test.

was not certified by peer review) is the author/funder. This article is a US Government work. It is not subject to copyright under 17 USC 1
and is also made available for use under a CC0 license.

FigS1

S1R overexpression rescues energy metabolism offset

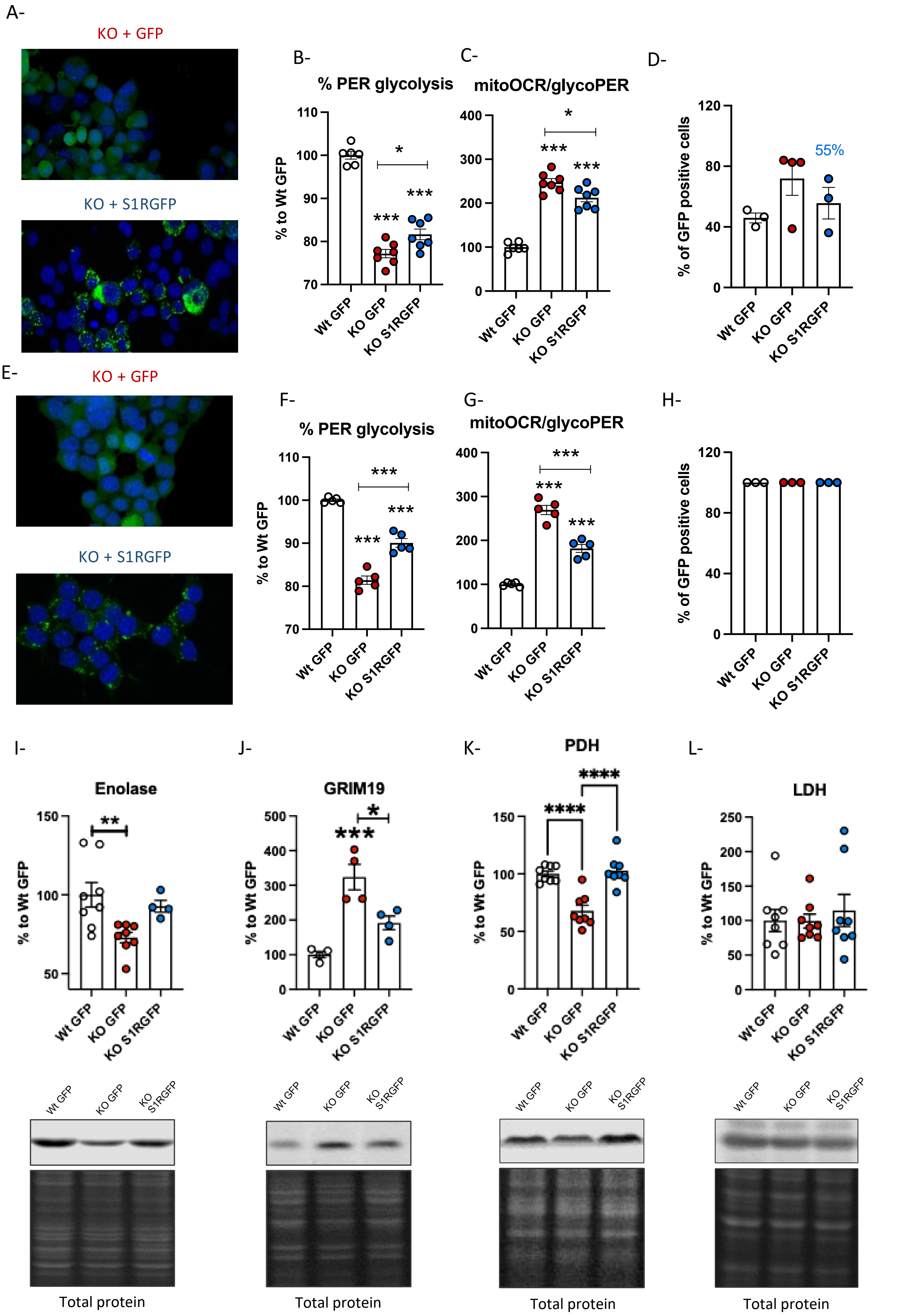

was not certified by peer review) is the author/funder. This article is a US Government work. It is not subject to copyright under 17 USC 1
and is also made available for use under a CC0 license.

FigS2

NAD/NADH-Glo™ and Peredox

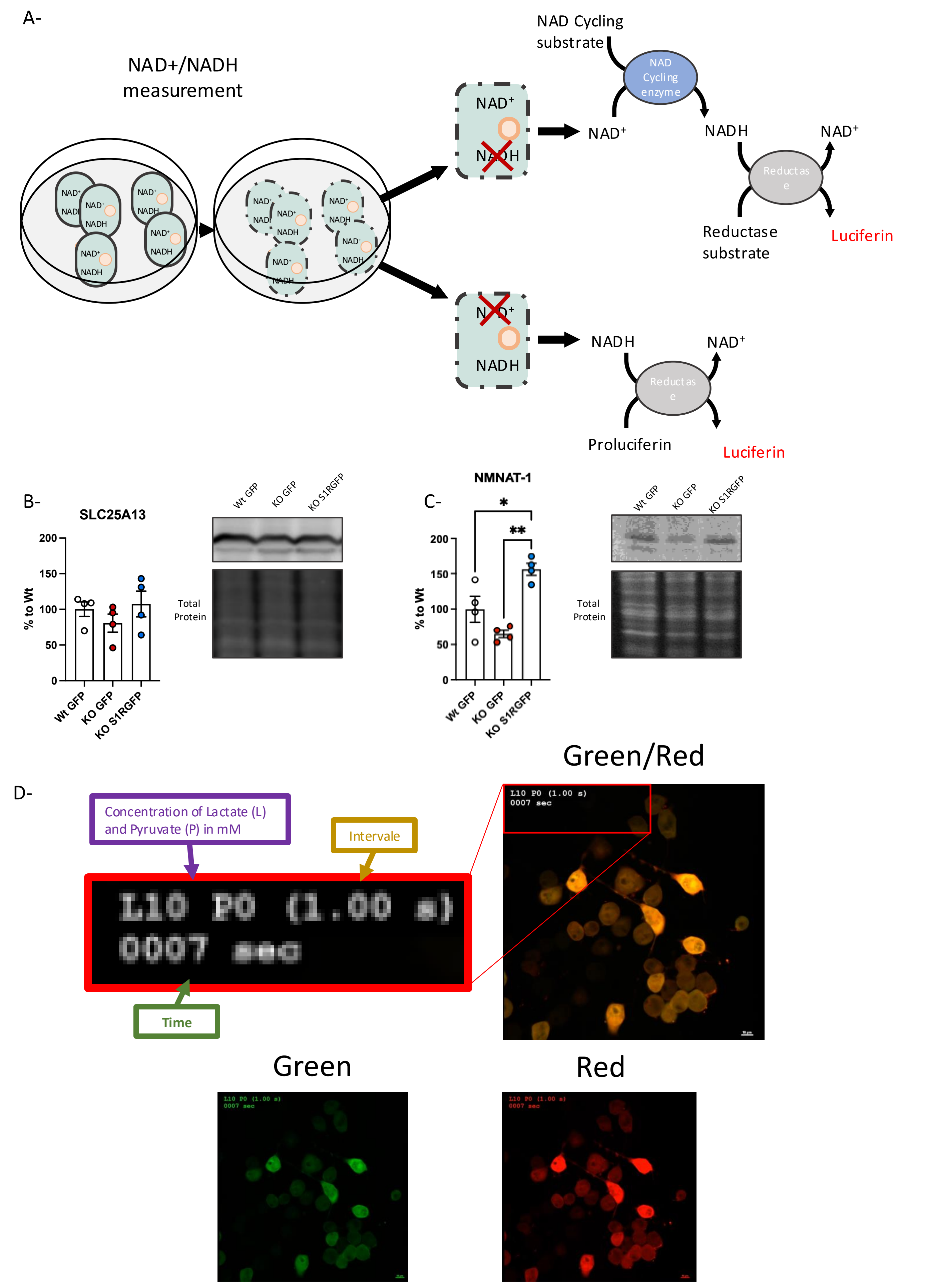

was not certified by peer review) is the author/funder. This article is a US Government work. It is not subject to copyright under 17 USC 1
and is also made available for use under a CC0 license.

FigS3

Supplementary figure 3

A-

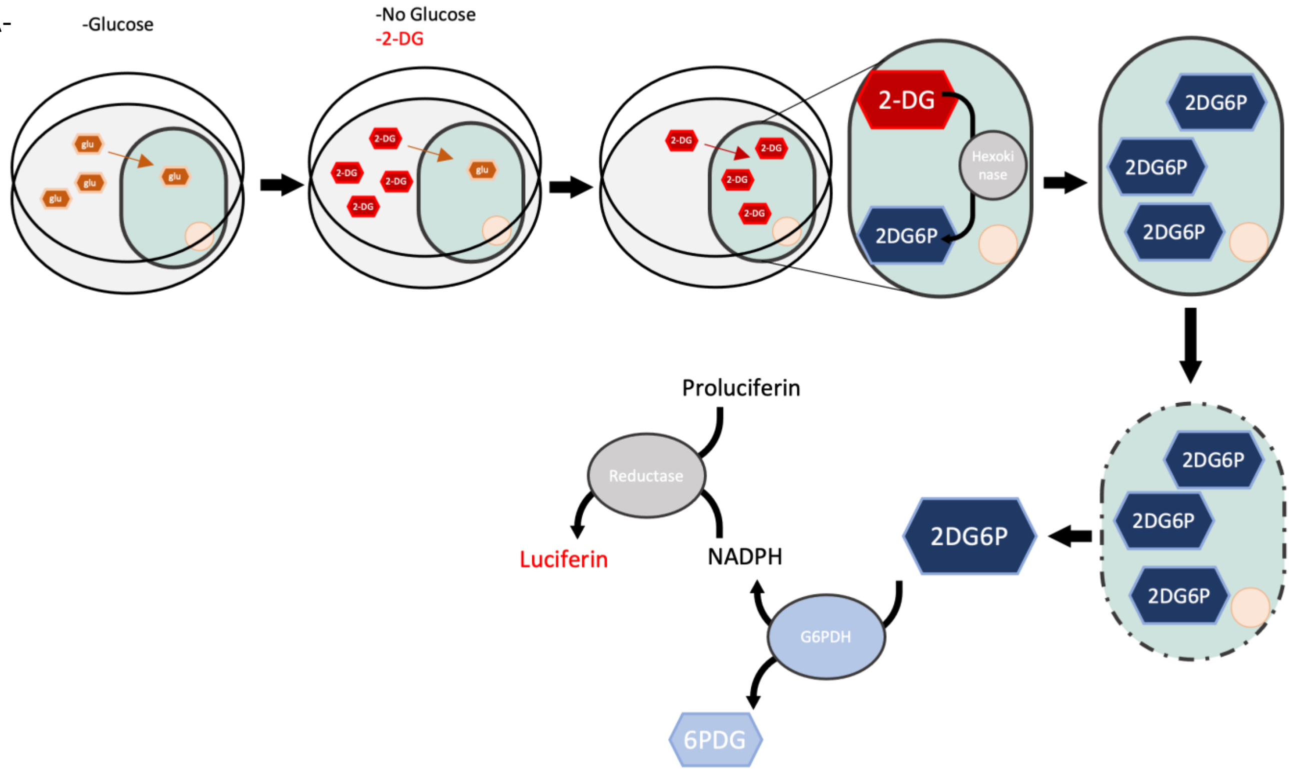

was not certified by peer review) is the author/funder. This article is a US Government work. It is not subject to copyright under 17 USC 1
and is also made available for use under a CC0 license.

FigS4

Supplementary figure 4

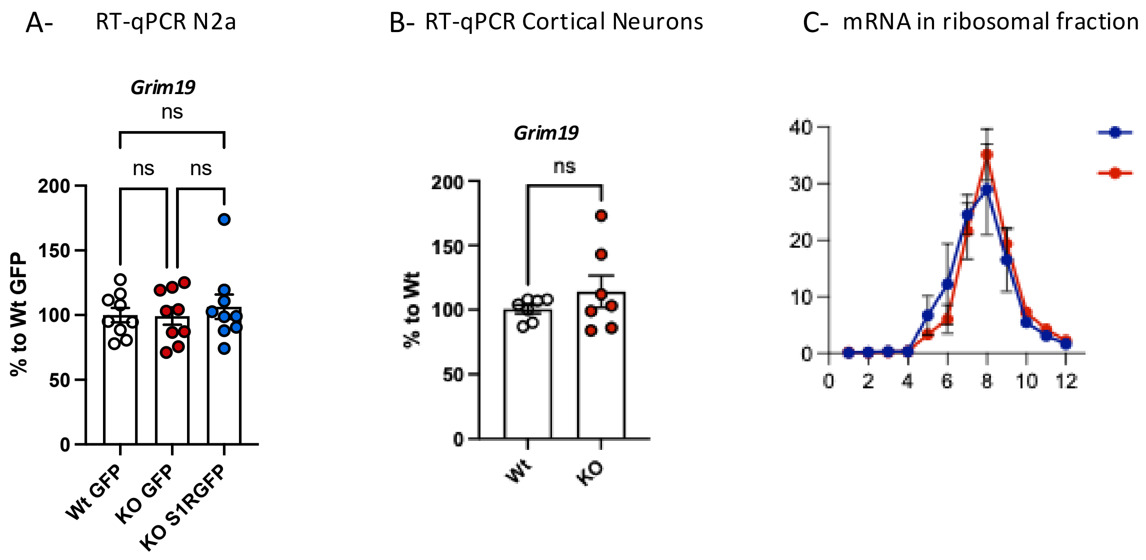

Supplement: Supplement 2 [file NIHPP2025.07.28.667250v1-supplement-2.pdf]
